# Discovering the world’s most endangered great whale species did not advance an issue-attention cycle in news media: Implications for Rice’s whale conservation and management

**DOI:** 10.1007/s13280-025-02265-y

**Published:** 2025-11-21

**Authors:** Marcus B. Reamer, Emily Yeager

**Affiliations:** 1 Department of Environmental Science and Policy, Rosenstiel School of Marine, Atmospheric, and Earth Science, University of Miami, 4600 Rickenbacker Causeway, Miami, FL 33149 USA; 2 Abess Center for Ecosystem Science and Policy, University of Miami, Coral Gables, FL 33146 USA

**Keywords:** Content analysis, Critical discourse analysis, Environmental communication, Issue-attention cycle, Marine conservation, Rice’s whale

## Abstract

Issue-attention cycles (IACs) follow the predictable rise and fall of media and public attention to topics through five defined stages. Using content analysis and critical discourse analysis, we analyzed 35 newspaper texts (2021–2024) about the Rice’s whale, a newly discovered and Critically Endangered species exclusive to the Gulf of Mexico. We investigated whether this discovery was enough to advance an IAC and found that, while Rice’s whale science, conservation, and policy has the elements of a topic likely to undergo an IAC, it remains in the first stage of the IAC with limited media attention and a focus on regional stakeholders and policy debates. Comparing this case to the North Atlantic right whale IAC (2010–2024), we offer insights for scientists, professionals, and advocates to prepare for potential future media attention and conservation conflict. Our findings highlight the importance of strategic communication and media analysis to conservation.

## Introduction

Human communication and media are essential components of socioecological systems (SES) as well as comprehensive conservation strategies and system-level change efforts (Brüggemann et al. 2023). Journalists, editors, and news organizations are important and influential actors in SES and environmental politics due to the accessibility, reach, and nature of the medium and their industry’s norms and practices (Boykoff 2009). These human actors are not outside observers of SES, but active and influential participants in them as they produce mediated representations of environmental and social problems (Putnam and Shoemaker 2007; Shoemaker and Reese 2014; Reamer et al. 2024). For that reason, news media coverage is not a collection of one-off texts; editorial gatekeepers determine which topics make it into public view (McCombs and Shaw 1972) and the body of coverage creates a discourse that shapes the knowledge and social realities related to those topics (Boykoff 2009). News media can make science accessible to non-expert publics, create a political arena where individuals and organizations compete for space and representation to shape public discourse, individual and organizational behavior, and influence decision-making, all of which can have material effects on species, habitats, and ecosystems (Hilgartner and Bosk 1988; Holtzhausen and Zerfass 2015; Takahashi and Tandoc 2016; Sachsman and Valentini 2020). This makes the study of human communication and media and their role in conservation and SES essential.

Biodiversity loss and anthropogenic climate change are paired challenges that must be addressed together, but climate change receives much more media and scholarly attention (Verissimo et al. 2014; Farber 2015; Geschke et al. 2023). Marine and coastal topics are especially underrepresented as they are particularly complex and challenging for people to understand and connect with, and for scientists, journalists, and practitioners to communicate with non-expert audiences (Schuldt et al. 2016; Kolandai-Matchett et al. 2021). This is in part due to real and psychological distance between daily life and aquatic environments (Carmi and Kimhi 2015; Schuldt et al. 2016; Kolandai-Matchett et al. 2021; Tang and Chooi 2023). Environmental professionals working in communication and outreach capacities often have to make decisions guided by “personal intuitions, individual preferences, and imitations of existing campaigns” rather than evidence-based best practices for strategic environmental communication (Liang et al. 2018, p. 135). This is especially true for communicators focused on marine and coastal topics because they are “hampered by the limited empirical evidence about what motivates their target audiences to engage” (Waldo et al. 2025, p. 2). There is a need for research that bridges these gaps and helps practitioners understand, design, and deploy communication strategies and tactics that work within existing media systems to effectively make marine and coastal issues feel accessible, personally relevant, and important to  their audiences (Vincent 2011; Thompson-Saud et al. 2018; Kolandai-Matchett and Armoudian 2020). There is also a need to work across sectors and stakeholders that include scientists, environmental professionals, journalists, and news editors to improve the flow of information and coverage about marine and coastal science, conservation, and management (Kolandai-Matchett et al. 2021; Reamer 2022a; Pinto and Matias 2023; Downing 2024). This study aims to address some of these gaps by exploring newspaper coverage of a recently discovered species of baleen whale, offering insights specific to this case and for conservation scientists, professionals, and advocates working on other topics in other geographies.

### Marine mammals and human society

Marine mammals and their conservation have benefited considerably from human communication and media, so much so that they are considered flagship species for the modern conservation movement (Reiss et al. 2006; Mast et al. 2014; Nelms et al. 2021; Reamer 2024). These animals—cetaceans in particular—have been a source of fascination and admiration to humans throughout recorded history (Mazzoldi et al. 2019; Brito et al. 2019). They have been represented through story and visual representations as sea monsters, divine beings, and even saviors for humans in distress, as well as nuisances who interfere with fishing or valuable commodities that can sustain entire communities (Mazzoldi et al. 2019).

Technological advances in shipping and whaling equipment allowed for unprecedented and unsustainable whaling activity that decimated stocks worldwide, with United States (US) whalers going so far as to drive the Atlantic gray whale (*Eschrichtius robustus*) to extinction in the mid-18th century (Linquist 2000; Garrison et al. 2019). As whale stocks throughout the global ocean declined to alarmingly low levels during the Industrial Revolution, the international community took notice and began monitoring and managing stocks to ensure the continued viability of the industry (Peterson 1992; Clapham 2016). As industrial whaling collapsed in the early half of the  20th century, attitudes about cetaceans began to change in western nations (Kalland 2009; Mazzoldi et al. 2019).

The 1950s were a pivotal decade as the first organized whale watching excursions launched as a non-consumptive use of whales, allowing people to witness these animals in their habitats firsthand, learn about their biology, behavior, and conservation, and develop emotional connections to them (Hoyt and Parsons 2014). Other science and environmental media like Roger Payne’s humpback whale song recordings, the Flipper TV show, and Greenpeace’s “Save the Whales” campaign allowed cetologists and advocates to connect non-expert audiences to cetaceans (Reiss et al. 2006; Reamer 2022b). As cetologists gained influence in the policy arena and citizens around the world developed greater empathy for these animals, policymakers responded with legal protections dedicated to the conservation and preservation of these species, most notably the US Marine Mammal Protection Act of 1972 (MMPA) that explicitly mentions the special and intrinsic value of cetaceans and other marine mammals to the nation (Reamer 2022b). Several species including the Eastern North Pacific gray whale (*Eschrichtius robustus*) and the humpback whale (*Megaptera novaeangliae*) successfully recovered as the result of dedicated legal protections and conservation investments, though they remain vulnerable to anthropogenic threats and environmental changes (Reynolds III et al. 2009; Bejder et al. 2016; Noad et al. 2019; Zerbini et al. 2019; Reamer 2022b).

Following decades of conservation marketing, non-expert publics have developed and maintained high regard for marine mammals, even if they do not have a strong understanding of these species or the complexities of their conservation and management (Kellert 1985; Scott and Parsons 2005; Naylor and Parsons 2018; Giovos et al. 2019). When they are in distress, injured, or deceased, marine mammals can attract public attention (Jahoda et al. 2025) and elicit an “exaggerated human behavioral response” (Bossart 2011, p. 676). The discovery of the Rice’s whale (*Balaenoptera ricei*) in 2021, a new, Critically Endangered baleen whale species found exclusively in the Gulf of Mexico (hereafter the Gulf), offers a unique opportunity to address gaps in the literature related to the role of mass media in marine conservation.

### Rice’s Whales

Through a combination of genetic and morphological analyses, Rosel et al. (2021) confirmed that a population of whales exclusive to the Gulf that had previously been thought to be a subspecies of Bryde’s whale (*Balaenoptera brydei*) is in fact a distinct lineage. Rosel et al. (2021) proposed naming this population of around 50 baleen whales (Hayes et al. 2020) the Rice’s whale, after marine mammal biologist Daniel Rice who first described the population in 1965 (Rice 1965). The Rice’s whale is most strongly identified by its unique mitochondrial genome but can also be distinguished from other baleen whales by three ridges on its rostrum and from other Bryde’s-like whales by its physical size and nasal bone structure (Rosel et al. 2021).

Recognized as the Gulf’s only resident species of baleen whale (Jefferson and Schiro 1997), the Rice’s whale is known to inhabit a very small portion of the northeastern Gulf near the coastlines of Alabama and Florida's panhandle year-round, including along De Soto Canyon (Garrison et al. 2024; Soldevilla et al. 2024; Wilcox Talbot et al. 2025). However, sightings and historical records of baleen whale presence in the northwestern and southwestern Gulf near Mexico may indicate Rice’s whale presence (or  prior presence) in a more expansive home range (Garrison et al. 2024; Soldevilla et al. 2024; Wilcox Talbot et al. 2025). As both the estimated population of Rice’s whales and their known habitat are small, the future of this population is particularly vulnerable to reproductive challenges and low genetic diversity alongside external threats including vessel strikes, ocean noise, marine debris, changes to prey distribution, fishing gear entanglement, anthropogenic climate change, oil and gas development, and oil spills (Garrison et al. 2024; NOAA 2025; Wilcox Talbot et al. 2025).

Prior to its recognition as a unique species, the Rice’s whale (then known as the Gulf of Mexico subpopulation of Bryde’s whale) was listed as Critically Endangered on the IUCN Red List (Corkeron et al. 2017) and received legal protections under the US Endangered Species Act (ESA) and the MMPA (NOAA Fisheries 2025). Since its reclassification, the Gulf population of Bryde’s-like whales that received legal protections alongside other Bryde’s-like whales has been re-listed separately as the Rice’s whale, both as Critically Endangered on the IUCN Red List (Rosel et al. 2022) and as Endangered under the ESA (NOAA Fisheries 2025). Given the Rice’s whale population size, its highly human-impacted and politically relevant habitat, its national and international conservation status, and that scientists still know relatively little about its life history, the Rice’s whale discovery presents an interesting and early opportunity to better understand the role of communication and media representation in shaping conservation outcomes.

### Theoretical framework: Issue-attention cycles

**Fig. 1:**
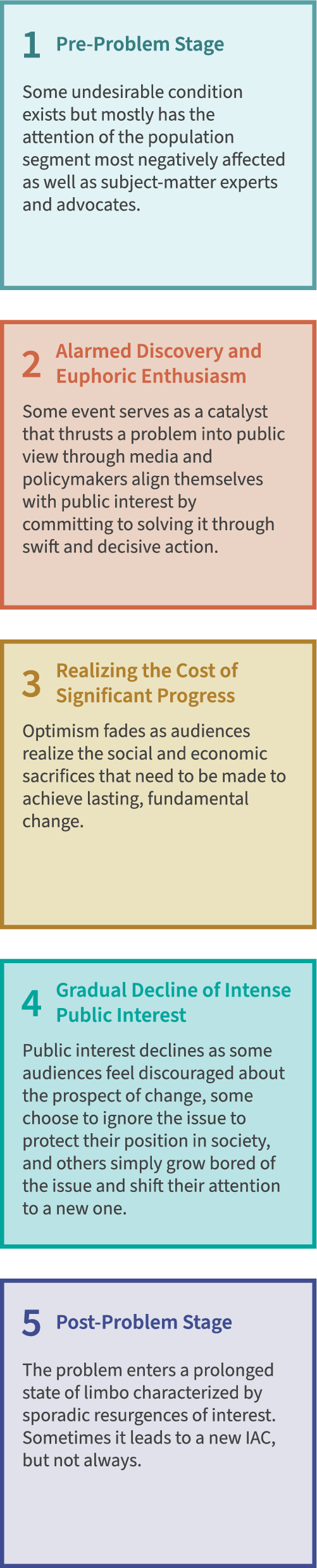
A graphic representation of the five distinct stages of Downs’ issue-attention cycle with defining characteristics of each (from Reamer and Rivera 2025)

Anthony Downs (1972) used the rise of the modern American environmental movement in the 1960s to argue for and test his issue-attention cycle (IAC) framework. This framework asserts that news media coverage follows predictable patterns to attract and sustain public attention for as long as possible before news organizations and audiences move on to more novel topics (Downs 1972). IACs are defined by five key stages: *pre-problem, alarmed discovery and euphoric enthusiasm, realizing the cost of significant progress, gradual decline in public interest*, and *post-problem* (Fig. 1). IACs play out across varying timelines and some repeat stages two and three multiple times before public and editorial interest begins to wane (Petersen 2009). Authors like Neuman et al. (2014) and Lörcher and Neverla (2015) have expanded upon Downs’ work to suggest that IACs are more about dynamics rather than finite cycles, meaning some broader topics like anthropogenic climate change or biodiversity loss can remain on the public agenda indefinitely. What the IAC framework makes clear is that no single social or environmental topic can sustain intense public attention forever, which has implications for strategic communication, policy engagement, and public problem-solving, especially for issues that require sustained attention and investment over long time scales.

Just because a topic is represented in news media does not guarantee it will eventually advance through the IAC. This is because of multiple factors like finite space and coverage capacity, media norms, and audience preferences, which together can create disparities between issues that are of high importance to scientists, experts, and advocates and of high interest to non-expert publics (Legagneux et al. 2018; Tiller et al. 2019; Reamer et al. 2024). In addition to creating a marketplace of ideas, news media also function as political arenas where organizations and coalitions compete to have their preferred topics represented, which then allows them to shape narratives and public discussions about social and environmental subjects using strategic communication tools and practices (Hilgartner and Bosk 1988; Holtzhausen and Zerfass 2015).

Downs (1972) outlines three characteristics of the public problems that are the most likely to advance through the IAC: who the problem affects most, the problem’s source, and the problem’s excitement. For issues that do progress through the IAC this means that the problem of interest affects some minority population—either numerically or relating to identity groups—while the majority benefits from the status quo; the problem is the result of some social arrangement that benefits the majority group; and the problem has no intrinsically exciting properties (Downs 1972). Examples of environmental topics that have been studied using the IAC framework include anthropogenic climate change (McComas and Shanahan 1999), energy conservation (Conrad 1987), plastics pollution (Bailey 2022), right whale science, conservation, and policy (Reamer et al. 2024; Reamer and Rivera 2025), and even metacycles about environmental stewardship and sustainability, which Djerf-Pierre (2013) defines as “the major fluctuations in attention to the entire domain of environmental issues over time” rather than the single-topic focus of IACs (p. 496).

To maintain audiences’ attention, journalists and editors often leverage cognitive biases by focusing on the negative aspects of a story, which lead consumers to pay closer attention to the issue and shapes how they learn and share information about it (Baumeister et al. 2001; Bebbington et al. 2017; Soroka et al. 2019). During an IAC, journalists and editors keep their audiences engaged by simplifying, problematizing, and dramatizing topics until these elements are exhausted, at which point both media producers and consumers inevitably move on to more novel and exciting topics (Downs 1972). Doing so creates a feedback loop between audiences and news organizations as audience preferences influence reporting and vice versa (McLuhan 1964). While these cycles serve the business model of journalism that relies on advertisements and subscriptions for revenue, they are not always congruent with the timelines, approaches, and goals of social and environmental change efforts that require sustained effort and attention (Reamer et al. 2024). Additionally, policy action and organizational change are the most likely at or around the peak of public attention and are more difficult to achieve later in the IAC, meaning the emergence of an IAC creates a limited window of opportunity to use communication and media to support change (Hilgartner and Bosk 1988; Reamer et al. 2024; Reamer and Rivera 2025). These are just some of the reasons that conservation scholars, professionals, and advocates can create stronger, more comprehensive conservation strategies by understanding the social function of communication and media, researching media coverage beyond counting the mentions of an organization or topic, and making evidence-based decisions to navigate a complex and fragmented media environment.

For conservation topics that do advance through an IAC, more coverage and public attention does not necessarily equate to better or even desired outcomes (Reamer et al. 2024). As Nisbet and Huge (2007) show, increased public interest moves issues from more exclusive and controlled environments into public forums, inviting new voices into the discourse and creating opportunities for changes in power dynamics. Given the difficulty in predicting or identifying when they emerge and why, and the many externalities that communicators cannot control for, IACs are challenging to navigate even when using best practices in strategic communication (Bailey 2022). This is especially true in cases involving oppositional industry or community groups that politicize issues and leverage scientific uncertainty to influence the discourse by using strategies like manufactured uncertainty or disinformation campaigns, which can be difficult to counter (Lupia 2013; Boan et al. 2018; Cook 2022; Maani et al. 2022). The journalistic medium and industry norms can even facilitate the use of these tactics. For example, the journalistic norms of balanced coverage and emphasis on conflict can also lead non-expert audiences to doubt expert consensus, further challenging conservation and sustainability efforts (Merkley 2020). Journalists and editors, even those knowledgeable about the science and politics of environmental issues they cover, “face challenges in exposing manufactured doubt to the public when it runs counter to public ideology, and unfortunately many media platforms do not allow for in-depth analyses” (Goldberg and Vandenberg 2021, p. 7). It is important for environmental communicators and organizations to understand these cycles and learn to both identify and navigate them to the best of their abilities if they aim to make progress on the topics they consider priorities.

### North Atlantic right whale science, conservation, and policy in media

**Fig. 2:**
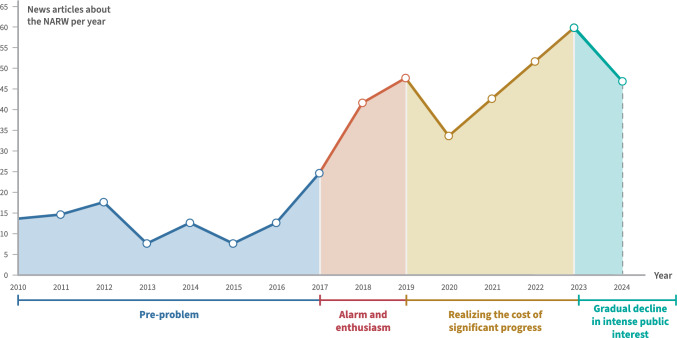
Progression of the NARW IAC from 2010 through 2024 (from Reamer and Rivera 2025)

Hunted to near extinction during the industrial whaling era and considered a species in crisis for decades, North Atlantic right whales (*Eubalaena glacialis*; NARW) are considered one of the world’s most endangered species of baleen whale with an estimated population of 372 individuals (Kraus et al. 2005; Pettis and Hamilton 2024). In the summer of 2017, an IAC about NARW science, conservation, and policy (SCP) advanced from the pre-problem stage alongside an Unusual Mortality Event (UME) that led to the death of at least 12 whales in Canadian waters, which, at the time were considered atypical of the species’ range (Reamer et al. 2024). From 2010 through 2024 that IAC progressed to its fourth stage, gradual decline of intense public interest, with the most intense public and media attention lasting from 2017 to 2023 (Downs 1972; Reamer et al. 2024; Reamer and Rivera 2025; Fig. 2).

The NARW IAC remained in the pre-problem stage from at least 2010 to 2017, when it advanced to stage two—alarmed discovery and euphoric enthusiasm—alongside the emerging UME (Reamer et al. 2024). As governments and stakeholders pledged to act swiftly and decisively to prevent further losses of NARWs along the species’ migratory range, media began focusing more intensely on the social and economic costs of policies that affected the American lobster fishery in New England (Reamer et al. 2024). By 2019 the NARW IAC progressed to stage three, realizing the costs of significant progress (Reamer et al. 2024). The news media discourse, led by *The Boston Globe* and reporter David Abel*,* became almost entirely focused on a high stake, winner-takes-all conflict between whale experts and advocates and lobster fishers and their allies, placing less emphasis on other threats to the species like vessel strike injuries and climate and environmental change, and using terms like battle, maelstrom, and war to describe the saga (Reamer et al. 2024). Both of these human actor groups were considered to be influential and powerful stakeholders in the region and the media reporting created a story of resistance as each sought to influence public discourse and decision-making by the National Marine Fisheries Service (NMFS) under the US Department of Commerce’s National Oceanic and Atmospheric Administration as the agency considered new and stricter fishing regulations designed to reduce the risk of fishing gear entanglement for NARWs (Hodgson et al. 2022; Reamer et al. 2024). Other important forms of media were released during this stage of the IAC, including the first documentary films focused on NARW SCP, *Entangled* (2020, dir. David Abel) and *Last of the Right Whales* (2021, dir. Nadine Pequeneza), which was later modified for public broadcasting and re-titled *Saving the Right Whale* (2023, dir. Nadine Pequeneza) (Reamer et al. 2023).

In December 2022, the US Congress and then President Joe Biden passed and signed a $1.7 trillion omnibus spending bill to prevent a partial government shutdown that included language blocking NMFS from implementing new regulations on the American lobster fishery until December 2028, a turning point in NARW conservation and management, (Reamer et al. 2024). This action facilitated the IAC's progression  into the fourth stage, gradual decline in intense public interest (Reamer and Rivera 2025). Following reactions from stakeholder groups in early 2023, both journalists and readers appeared to generally move on to other topics as the conflict between whale experts and advocates and lobster fishers and their allies de-escalated (Reamer and Rivera 2025). This, despite other threats like vessel strikes and climate and environmental change continuing to place pressure on the NARW population and needing to be addressed (Reamer and Rivera 2025). NARW SCP still receives coverage in the cycle’s later stages, but it is more episodic, focusing on individual whale sightings, births, injuries, and deaths, and there is likely more editorial resistance in determining which updates are considered newsworthy and receive representation in coverage (Reamer and Rivera 2025).

The previous analyses of the NARW IAC showed that the threat of fishing gear entanglement in the New England region was likely the easiest for journalists to simplify, dramatize, and problematize for their readers because the problem was visible, it involved popular and recognizable marine resources—whales and lobsters—and it was an active and escalating conservation conflict where the stakes were high for human actor groups representing two ideological sides of the issue (Reamer et al. 2024; Reamer and Rivera 2025). While research on IACs across a range of topics can offer valuable lessons that Rice’s whale scientists and advocates can apply to their own work, the NARW IAC allows for direct comparison because it involves another Critically Endangered whale species in the US and has already progressed through most of the IAC’s five stages (Reamer and Rivera 2025).

## Research objectives

Through this study, we aim to contribute to the understanding of the social and governance dimensions of Rice’s whale conservation and management and offer informed insights about the role of news media in conservation science and practice. Specifically, our objectives are to: (1) understand at which stage the IAC for this topic is from the species’ recognition in 2021 to 2024; (2) compare these results to previous research on the IAC about NARW SCP; and, (3) offer informed insights to scientists, professionals, and advocates working on Rice’s whale SCP regarding the use of communication and media to support the social and governance dimensions of their work, and that conservation scholars, practitioners, and advocates working on other topics can learn from and apply.

## Materials and methods

Media can be studied across three distinct stages: (1) how and why they were created by media producers, (2) the format and the messages contained within them, and (3) their effects on audiences (Painter 2021). This exploratory study focuses on stage two—the media and their messages—using a social listening approach (Staddon et al. 2023). To understand media coverage about Rice’s whale SCP from its recognition as a distinct species in 2021 through 2024, we used complementary content analysis (CA) and critical discourse analysis (CDA) to analyze sampled texts (Hardy et al. 2004). Where CA would reveal patterns in the coverage—like leading publications, rates of coverage per year, and common topics—CDA would explore the language in the texts co-created by journalists, their sources, and editors to understand the social context of Rice’s whale SCP, including stakeholder perspectives and values, areas for potential conflict and dramatization, relevant government institutions and legal frameworks, and the distribution of political power between human actor groups. The combined results offer a richer understanding of the social and governance dimensions of Rice’s whale conservation and the role that news media has and will continue to play in it than if these methods were used independently.

We used ProQuest’s Global Newsstream database (formerly Newsstand) to search for sampled texts. Our query was for texts including the term Rice’s whale or Rices whale and was delimited to articles and opinion–editorial letters (op-eds) that were published in any newspaper in the database between January 1, 2020, and December 31, 2024. We intentionally set the range of publication dates one calendar year before Rosel et al. (2021) called for the official recognition of Rice’s whales as a distinct species. This was in case there were any op-eds or other texts on the subject that may have been published ahead of the peer-reviewed journal publication reporting on the new discovery.

The search initially yielded 54 outputs, all from January 2021 onward and all of which were in English. After removing duplicate results, 46 texts remained. We used a codebook to capture data from the sampled texts. The codebook was a version of the one used in previous studies that explored the IAC focused on NARW SCP (Reamer et al. 2024; Reamer and Rivera 2025). Harrison and Loring’s (2020) transdisciplinary framework for understanding conservation conflicts through story informed the codebook’s design and approach to analysis. We used a grounded theory approach (Charmaz 1995) to update the codebook based on a first reading of all texts in the sample for this study. Author MR coded all 46 texts in order from oldest to newest, removing 11 additional texts because they were word-for-word reprints of the same text in multiple newspapers but published under unique headlines. The final sample for analysis included 35 texts. We engaged directly with our data during analysis; at no point did we use a large language model like ChatGPT or Claude.

## Results

### Content analysis

**Fig. 3:**
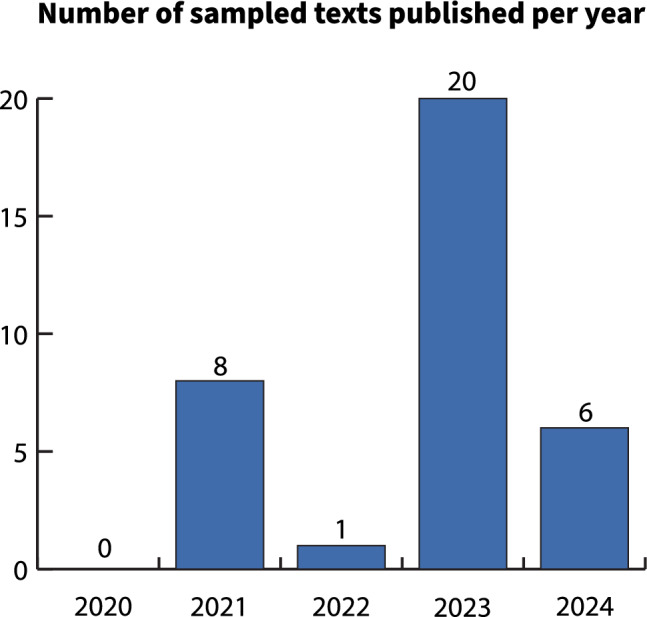
Annual rate of coverage in the sampled texts

We analyzed 35 unique newspaper articles and op-eds about Rice’s whale SCP using complementary CA and CDA methods. None of the sampled texts were published before January 2021, when Rosel et al. (2021) was published. From January 1, 2021, to December 31, 2024, the annual rate of coverage about right whales ranged from one (2022) to 20 (2023) texts (Fig. 3). Nearly all the included texts were standard articles (*n* = 31, 88.6%), though three op-eds and one longer-form feature article were included. More than 77% of all texts were published in 2023 and 2024 (*n* = 27), with 57% of all sampled texts published in 2023 (*n* = 20).

Nineteen distinct newspapers published the analyzed texts. Most were published in US newspapers (*n* = 25, 71.4%), with some international coverage representing newspapers from the U.K. (*n* = 6, 17%), China (*n* = 2, 5.7%), Lebanon (*n* = 1, 2.9%), and Sri Lanka (*n* = 1, 2.9%). Of the 25 texts published in US newspapers, more than 75% (*n* = 19) were from newspapers in the Gulf states of Florida (*n* = 16), Louisiana (*n* = 1), Mississippi (*n* = 1), and Texas (*n* = 1). *The Pensacola News Journal* (FL) was the leading publication (*n* = 9), followed by *The Tampa Bay Times* (FL) (*n* = 5), *The New York Times* (NY) (*n* = 3), and *The News Press* (FL) (*n* = 2).

**Fig. 4:**
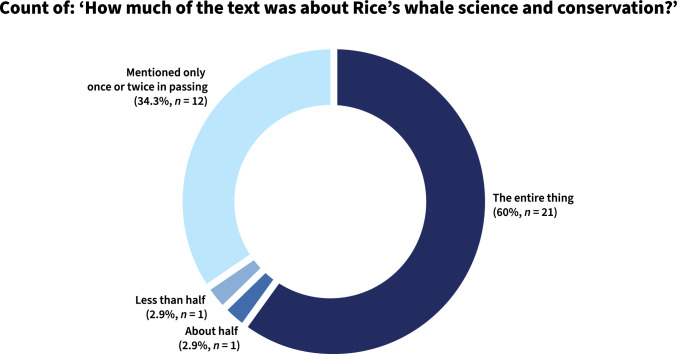
Proportion of texts and their focus on Rice’s whale science, conservation, and policy

**Fig. 5:**
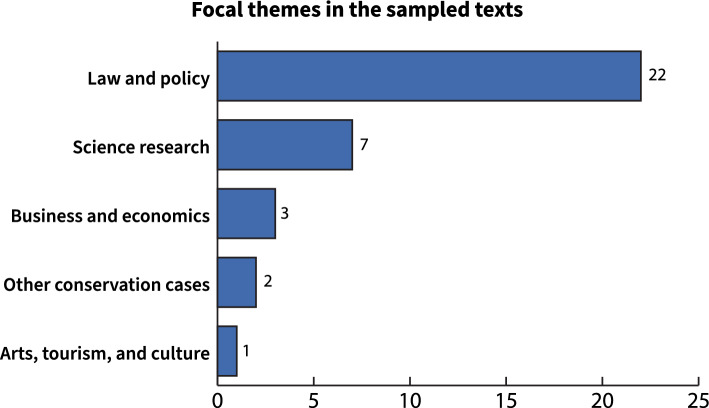
Frequency of text associated with each of the five identified themes

There were 24 unique authors with attributed bylines, five of whom (20.8%) published more than one text in the sample (Table 1). Journalist Tom McLaughlin was the most prolific author in the analyzed coverage, producing nearly one-quarter of the  texts (*n* = 8), as many as the next four other authors with multiple bylines combined (Table 1). 60% of the texts were exclusively about Rice’s whale SCP, and a little more than one-third only mentioned the species in passing within a text about some other topic (Fig. 4). Nearly two-thirds of the sampled texts focused on law and policy themes (*n* = 22) and one-fifth focused on science research, either published or ongoing (*n* = 7) (Fig. 5).

**Table 1:** Authors with multiple bylines in the sample and the newspaper(s) their work appeared in

Author	Publication(s)	Count
Tom McLaughlin	*Pensacola News Journal* (US, FL) *Tampa Bay Times* (US, FL) *The News Press* (US, FL)	8
Max Chesnes	*Tampa Bay Times* (US, FL)	2
Collin Bestor	*Pensacola News Journal* (US, FL)	2
Lisa Friedman	*The New York Times* (US, NY)	2
Bethany Blankley	*The Times Tribune* (US, PA) *The Republican Herald* (US, PA)	2

### Critical discourse analysis

In our reading of the 35 texts, we identified four key topics: the discovery and designation of the Rice’s whale as a distinct and Critically Endangered species exclusive to the Gulf; a petition to NMFS to implement a vessel speed reduction rule and nighttime shipping ban in the northern Gulf; efforts in the US Congress to pass legislation that would exempt the US Air Force at Eglin Air Force Base in the Florida panhandle from ESA and MMPA requirements pertaining to military testing activities that could produce significant ocean noise; and litigation related to federal oil and gas lease sales in the Gulf region. These four topics represented nearly 83% of the sampled texts (*n* = 29). This section explores the discourse surrounding each of these four topics in detail. We present the topics in the order in which they first appeared in the sample, and our analysis includes the environmental and policy events featured, the human actor groups involved and their relationships and viewpoints, and, where appropriate, representative quotes (Table 2).

**Table 2:** A table listing representative quotes and excerpts from the sampled coverage for each of the four key topics in the discourse

Topic	Document #, Publication (Country, State)	Journalist	Quote/Excerpt	Stakeholder affiliation
*Discovery and recognition*	40, *The Guardian* (UK)	Joshua Rapp Learn	“I was surprised that there could be an unrecognized species of whale out there, especially in our backyard,” said Lynsey Wilcox, a geneticist with the US National Oceanic and Atmospheric Administration who helped uncover the new species. “I never imagined I would be describing a new species in my career, so it is a very exciting discovery.”	*Federal agency*
	37, *The News Press* (US, FL)	Tom McLaughlin	“They would have historically ranged along the entire shelf, but they've been pushed to the Northeastern Gulf because it's the quietest part of the Gulf of Mexico,” [Florida/Alabama organizer for the environmental group Healthy Gulf, Christian] Wagley said. “All the drilling and shipping activity going on in the Central and Western Gulf is like a heavy metal concert to them.”	*Environmental groups*
	40, *The Guardian* (UK)	Joshua Rapp Learn	“They are the most endangered, or nearly the most endangered, baleen whales in US waters,” [biological oceanographer at Scripps Institution of Oceanography at the University of California San Diego John] Hildebrand says. “In terms of the responsibility for the health of the whale, it really does fall on us.”	*Scientists and researchers*
	46, *The Tampa Bay Times* (US, FL)	Zachary T. Sampson	The animal formerly known as the Gulf of Mexico Brydes whale could need a new name. The endangered whale may upon confirmation be an entirely new species, according to research from the National Oceanic and Atmospheric Administration. Scientists have long puzzled over the whales, with fewer than 100 estimated in existence, perhaps among the rarest in the world. Examination of a skeleton found off of Everglades National Park in 2019, which was later buried at Fort De Soto Park to decompose, has led to new clues	*Journalist*
*Vessel speed reduction rule attempts in the Gulf*	29, *Pensacola News Journal* (US, FL)	Tom McLaughlin	“The devil is in the details,” said [Florida/Alabama organizer for the environmental group Healthy Gulf, Christian] Wagley. “We have to try to hit a sweet spot to protect the whales without unduly burdening the fishing industry and boaters.”	*Environmental groups*
	24, *Pensacola News Journal* (US, FL)	Collin Bestor	“Our family, like many families, make their living on the Gulf,” said Anne Hensey, whose husband, Michael, is captain of the Jumping Monkey. “While we feel sympathetic to the cause of the Rice's whale, the limitation proposed by NOAA in the petition will cause undue stress on everything from commercial fishing… to recreational fishing.” She went on to tell the commission that if this proposal is enacted, it could be the final “nail in the coffin” for many of the local businesses that rely on fishing to make a living	*Recreational fishers*
	24, *Pensacola News Journal* (US, FL)	Collin Bestor	Parker Destin, who owns Dewey Destin's Seafood restaurant, echoed [Anne] Hensey's comments. He also noted that the fishing industry is part of Okaloosa County's heritage, and it needs to be protected so that it can continue	*Private business*
	23, *Pensacola News Journal* (US, FL)	Tom McLaughlin	[Santa Rosa County] Commissioner James Calkins said NOAA's proposal, backed by Healthy Gulf and other conservation groups, amounted to the federal government impeding the rights of American citizens. “It's disturbing our federal government would impose such a ban,” he said	*State and local legislatures*
	15, *Pensacola News Journal*, (US, FL)	Collin Bestor	“This is a great win for Destin's fishing industry, tourism, and the military, which would have been greatly affected if the vessel speed ruling had moved forward,” Ponder said in a statement. “I think that NOAA's observations and willingness to listen to the suggestions about education and outreach to protect the Rice's whale strikes the perfect balance between the environment and the needs of the military and tourism which is so vital in Okaloosa County.” Goodwin also praised NOAA for “restraint and wisdom by recognizing how this type of draconian regulation would have negatively affected the military, our economy, and everyday residents.”	*State and local legislatures*
	25, *Tampa Bay Times* (US, FL)	Max Chesnes	“Any useful information that industry, the public or the scientific community can provide to help us inform our decision-making would be really appreciated,” said [Grant Baysinger, a contractor with the National Marine Fisheries Service, which is currently accepting public comments]. That's really what we're looking for at this stage.	*Federal agencies*
	25, *Tampa Bay Times* (US, FL)	Max Chesnes	Should ships in the Gulf of Mexico slow down while crossing the habitat of one of the rarest whales on earth? That’s the question swirling around a new proposal to protect the roughly 50 Rices whales left in existence. Scientists and ocean advocates say it is a no-brainer: There's evidence that boats have struck and killed the whales. A speed limit and nighttime shipping ban in and around the whales habitat would curb deaths, they say. On the other side of the debate, Florida ports fear a speed limit and nighttime travel restriction would hinder the state's shipping industry, which saw record-high cargo last year. Industry leaders told the Tampa Bay Times they fear the state's port economy would be in danger, or even shut down, if the proposal was approved	*Journalist*
*Congressional efforts related to US Air Force activities and ocean nois*e	6, *Pensacola News Journal* (US, FL)	Tom McLaughlin	[Representative Matt] Gaetz [(FL-01)] defended his amendment by saying Eglin is not utilizing enough of its estimated 120,000-square-mile water range. “Since I have been in Congress, there has been no harm to Rice's whales from munitions and missile testing at the Eglin Gulf Test & Training Range. And Eglin Air Force Base does not believe reopening the areas currently restricted would endanger the Rice's whales,” he said in an email. “Therefore, my amendment is an effort to get the range fully operational again, which is vital to our national defense.”	*US Congress*
	6, *Pensacola News Journal* (US, FL)	Tom McLaughlin	“That Eglin [Air Force Base] would work as diligently as it did to help preserve Rice's Whale comes as no surprise”, said the National Resources Defense Council's Michael Jasny. “Eglin for decades has worked to serve as solid stewards of the environment,” Jasny said. “Real environmental leadership has been realized by Eglin.”	*Environmental groups*
	6, *Pensacola News Journal* (US, FL)	Tom McLaughlin	The whale's only chance for survival likely depends upon compliance with the provisions of the Endangered Species Act and Marine Mammals Protection Act, said Christian Wagley an organizer for the conservation group Healthy Gulf. “These laws work and the people of this nation support them, Congress doesn't need to meddle with them,” he said. Groups like Healthy Gulf question the proposed legislation because, as was the case last year, the Air Force did not request that Gaetz introduce it and it is unclear what benefits the Fort Walton Beach Republican believes can be derived from the exemption he seeks	*Environmental groups*
	34, *Pensacola News Journal* (US, FL)	Tom McLaughlin	[General Glen VanHerk] informed Gaetz that as a combat commander it would not be his role to work with him to see the test range opened to areas that include Rice's Whale habitat. A Department of Defense representative told Gaetz the information he had presented to the general would be studied	*Federal agencies*
	6, *Pensacola News Journal* (US, FL)	Tom McLaughlin	US Rep. Matt Gaetz didn't get his way last year when he sought to give Eglin Air Force Base personnel the right to fire munitions into the Department of Defense's vast Gulf of Mexico Test Range without considering the harm that might inflict on population of the Rice's Whale, one of the world's most endangered marine mammal species. Florida's First District congressman has again introduced legislation asking his colleagues to support Air Force exemptions to the Endangered Species Act and the Marine Mammals Protection Act	*Journalist*
*Litigation over federal oil and gas lease sales*	18, *The New York Times* (US, NY)	Lisa Friedman	Ryan Meyers, the senior vice president and general counsel at the American Petroleum Institute, said the Biden administration's attempts to hem in drilling jeopardized the country's energy security. “It should not take a court order or an act of Congress for [the US Department of the] Interior to carry out its responsibility to meet the energy needs of the American people,” he said	*Trade associations*
	1, *The Financial Times* (UK)	Myles McCormick and Jamie Smyth	TotalEnergies chief executive Patrick Pouyanné, highlighting the $60bn in different penalties BP was forced to pay [following the Deepwater Horizon oil spill in 2010]. The industry points out that safety measures have changed considerably since the accident, including sweeping improvements in spill control. “It's an event that forever shaped our company and, in fact, reshaped and changed the industry … not just in the Gulf of Mexico, but globally,” says Andy Krieger, senior vice president for BP operations in the region. “What is fundamentally different and changed is the mitigations in place and the management of that risk and the rigor with which that risk is managed.”	*Private business*
	17, *Hattiesburg American* (US, MS)	Colin Campo	“These baseline protections for the Rice's whale are quite literally the least we could be doing to save the species from extinction,” Earthjustice attorney Steve Mashuda said. “Meanwhile, the government is still enabling the oil industry to bid on 67 million acres of the Gulf. These oil companies are looking at the full glass after one sip and calling it empty”	*Environmental groups*
	14, *Yerepouni Daily News* (Lebanon)	Charles Kennedy	“The oil industry fought tooth and nail to tear up basic measures to save one of the most endangered marine mammals in the world,” George Torgun, an attorney with Earthjustice, told Bloomberg. “This could be the difference between doing the bare minimum to save this species and allowing it to vanish.”	*Environmental groups*
	10, *The News Press* (US, FL)	Tom McLaughlin	“One event, like an oil spill, could wipe out the species,” said John Ososky, manager of the marine mammal collection division of the Smithsonian Institute's Museum of Natural History. Ososky played a critical role in bringing about the 2021 declaration of Rice's whale as a unique whale species	*Scientists and researchers*
	18, *The New York Times* (US, NY)	Lisa Friedman	“There's a delicate dance here,” said Michael Gerrard, director of the Sabin Center for Climate Change Law at Columbia University. “There's a gap between what some advocates want the president to do, and what he can actually do, especially given a conservative Supreme Court, a hostile House of Representatives and a divided Senate.” Look no further than the tortured history of Lease 261, a 73 million acre tract of water in the Gulf of Mexico that is slated to be leased to oil companies next month	*Scientists and researchers*
	4, *The Tampa Bay Times* (US, FL)	Cara Fleischer	Whales get hit by boats as they bask at night near the oceans’ surface. Oil spills are another constant threat—BP’s 2010 Deepwater Horizon disaster killed an estimated 20% of their population. Today, the Gulf has about 2,000 oil and gas platforms and more than 20,000 miles of active pipelines. The fossil fuel industry has fought against protections for this species—including set asides that would create safe zones in whale habitat and common-sense speed limits to keep whales from getting run over	*Citizens (activist)*
	13, *The Times Tribune* (US, PA)	Bethany Blankley	[The Bureau of Ocean Energy Management] announced, “Pursuant to direction from the Court,” it will “include lease blocks that were previously excluded due to concerns regarding potential impacts to the Rice’s whale population in the Gulf of Mexico. BOEM will also remove portions of a related stipulation meant to address those potential impacts from the lease terms for any leases that may result from Lease Sale 261.”	*Federal agency*
	17, *Hattiesburg American* (US, MS)	Colin Campo	[US Federal District Judge James D. Cain Jr.] concluded that the last minute removal of the acreage was politically motivated. “This leaves the impression that the 2023 Record of Decision is merely an attempt to provide scientific justification to a political reassessment of offshore drilling,” he said. The process followed here looks more like a weaponization of the Endangered Species Act than the collaborative, reasoned approach prescribed by the applicable laws and regulations. “Even when an agency's decision is based on political considerations, it is not excused from justifying the position particularly when the decision is a pivot from a prior policy.”	*Federal courts*
	13, *The Times Tribune* (US, PA)	Bethany Blankley	Louisiana Attorney General Jeff Landry said the rulings were “a major win not only for the rule of law, but also for Louisiana jobs and affordable energy. At a time when working families are being squeezed by unaffordable Bidenomics, I am glad to deliver yet another victory defeating overreaching bureaucrats. …Congress is clear: lease sales must take place; so we are grateful the Judge cut through the noise and upheld the law.”	*State and local government agencies*
	7, *The New York Times* (US. NY)	Ivan Penn	The Biden administration had planned to scale back lease sales for oil drilling in the Gulf, which environmentalists said would help protect Rice’s whales. In August, the Bureau of Ocean Energy Management reduced the area available for leases from 73 million acres to 67 million acres	*Journalist*

#### Discovery and recognition

Rosel et al.’s (2021) genetic and morphological research that argued for the recognition of Rice’s whales as a distinct species exclusive to the Gulf attracted media attention in January and February 2021. Coverage of this topic represented 20% of the analyzed texts (*n* = 7); all of them were standard articles. The stakeholder groups included in these articles were overwhelmingly represented by the NMFS scientists and partners at Mote Marine Laboratory (FL) who helped retrieve the whale carcass that supported this research and at the Smithsonian Institution (US) and Japan’s National Museum of Nature and Science who helped to conduct and publish this pivotal study. External expert validators from other universities and environmental organizations were included in the form of offering comments about the research findings and implications. This discourse focused on the years of work leading up to the discovery, challenges associated with marine mammal research, how researchers were able to reach their conclusion, and what they hoped it would mean for the conservation of this so-called new and imperiled species. These seven texts emphasized research partnerships between organizations and across sectors (*n* = 6, 85.7%) and established urgency by mentioning the Critically Endangered status of this newly recognized species (*n* = 3, 42.9%). When speaking of the whales, experts leveraged patriotic framing to present the Rice’s whale as a uniquely American species due to its exclusivity to the Gulf and expressed optimism and hope that citizens, organizations, and governments would fulfill their responsibility to support its recovery (e.g., Learn 2021). Four of these texts (*n* = 57.1%) discussed threats to Rice’s whales, introducing multiple, interrelated threats under the umbrella term of an industrialized area of the ocean. Individually named threats included offshore oil and gas development (*n* = 3), vessel strikes (*n* = 3), ocean noise (*n* = 2), pollution and debris (*n* = 1), fishing gear entanglement (*n* = 1), warming ocean temperatures (*n* = 1), and a small population size (*n* = 1).

#### Vessel rule attempts in the northern Gulf

A petition to have NMFS implement vessel speed restrictions and a nighttime shipping ban in the northern Gulf to protect Rice’s whales received media attention beginning in October 2022. It began when Earthjustice petitioned NMFS on behalf of Healthy Gulf—a regional environmental organization that works in Texas, Louisiana, Mississippi, Alabama, and Florida—to implement new regulations for recreational and commercial vessels to protect Rice’s whales in the northern Gulf (McLaughlin 2022). Coverage about this topic lasted until November 2023 when NMFS rejected the petition and chose not to enact any new vessel speed rules in the area (Bestor 2023). There were seven unique texts about this topic over the course of the year, six of which were standard articles and one an op-ed by Healthy Gulf. Coverage of this topic represented 20% of the analyzed texts.

Discourse about this topic centered around the pros and cons of the proposed regulations, with a general focus on vessel speed restrictions and less emphasis on the nighttime shipping ban. Regional and national environmental groups like Healthy Gulf, Natural Resources Defense Council (NRDC; NY), Center for Biological Diversity (AZ), New England Aquarium (MA), and Defenders of Wildlife (Washington, DC) were in support of the rule. Supporters of the rule framed it as a common sense, win-win solution that required minimal disruption for people and businesses, one that would achieve reduced local air pollution along with the protection and recovery of the only whale population that is exclusive to their local Gulf waters and part of the region's maritime culture. County-level legislatures in the area like the Okaloosa County Board of County Commissioners (FL) and the Santa Rosa County Commission (FL), as well as Florida port authorities, recreational fishing associations like the Pensacola Big Game Fishing Club (FL) and the American Sportfishing Association’s Keep America Fishing (VA), and local seafood restaurants were apprehensive about the rule and its argued benefits to the Rice’s whale population. Opponents expressed an interest in protecting the whales but had concerns about the practicality of any vessel speed reduction requirements and the benefits they would provide the whales. They did so by questioning the accuracy and lack of data supporting environmental groups’ petition to NMFS for the vessel speed rule. Recreational fishers expressed concerns about unreasonable increases in travel times to and from fishing areas, with an emphasis on DeSoto Canyon located in the Gulf off the coast of Alabama and Florida. Their concerns related to the possible impacts these changes could have on annual trophy fishing tournaments that were economically important to the region, as well as local businesses that rely on fishing, like charter companies and restaurants. State port authorities cited economic costs associated with longer shipping times, and local governments framed the possible rule as federal overreach by NMFS and passed resolutions to formally oppose the petition. Additionally, the rule’s opponents sowed doubt about any benefits to the Rice’s whale population resulting from slower ship speeds by citing the population of about 50 individuals spread across a large area of water; they argued that the probability that vessels would ever cross paths with these whales was already incredibly low and the proposed rule was overly cautious. Where NMFS spokespeople and stakeholders engaged, the agency plainly expressed a commitment to fair process and legally compliant decision-making. Four texts about the vessel speed rule petition mentioned a public comment process that readers could participate in (57.1%), two of which included directions on where to find the petition online and how to participate. Though vessel strikes were the focus of texts about the vessel speed reduction rule, other threats to the Rice’s whale were mentioned as secondary, which included offshore energy development (*n* = 3) and ocean noise (*n* = 2).

#### Congressional efforts related to US Air Force activities and ocean noise

Legislative efforts by former US Representative Matt Gaetz (FL-01) that would affect Rice’s whales first gained media attention in March of 2023 (McLaughlin 2023) and again in May and July of 2024 (McLaughlin 2024a, 2024b). There were four unique texts about this topic which represented 11.4% of the sampled texts; all four were standard articles. The stakeholder groups mentioned in these articles included Members of Congress, the US Air Force, NMFS, and two environmental groups: Healthy Gulf and NRDC. This discourse focused on Gaetz’s efforts to include language in annual National Defense Authorization Act bills during markup in the House Armed Services Committee. Gaetz’s amendments would have exempted the US Air Force at the Eglin Air Force Base in the Florida panhandle from provisions in the ESA and MMPA related to permitted testing activities. These actions drew criticism from Healthy Gulf and NRDC, who questioned the representative’s motives and pointed out that NMFS already had the authority to permit incidental takes of marine mammals for national security purposes under the ESA and MMPA. The US Air Force at Eglin Air Force Base was also quoted in Committee hearings as not having requested these amendments to be introduced (Table 2). Healthy Gulf and NRDC also offered praise for the Eglin Air Force base as strong stewards of the environment and having historically taken steps to mitigate harms to marine mammals and other natural resources in their activities (Table 2). All four texts discussed the threat of ocean noise associated with military activities to the Rice’s whale population, and no other threats were mentioned.

#### Litigation over federal oil and gas lease sales

Decisions made by the Biden Administration relating to federal oil and gas lease sales, including in the northern Gulf, received media attention beginning in August 2023. There were 12 unique texts that referenced this topic, 10 of which were standard articles, and one each of a longer-form feature article and an op-ed. Coverage of this topic represented 31.4% of the sampled texts. Reporting on this topic followed attempts from the Biden Administration to place a moratorium on oil and gas lease sales on federal lands and in federal waters, including Lease 261, an area in the northern Gulf that the Administration reduced in size as part of a settlement agreement with the environmental nonprofit, Sierra Club. Following an initial op-ed in *The Wall Street Journal* in August 2023 opposing the Biden Administration’s moratorium (Editorial Board 2023), texts about this topic largely followed legal challenges, court decisions, and stakeholder reactions to them, with Rice’s whales usually mentioned in passing as an endangered species that federal agencies used to justify decision-making about oil and gas leases in the northern Gulf.

The human stakeholders in these texts included whale experts and advocates, oil and gas interests and government officials from Gulf states with robust fossil fuel development industries, federal agencies, and federal courts. Whale experts and advocates most commonly included the regional environmental group Healthy Gulf with support from national environmental organizations: Earthjustice (CA), NRDC, Center for Biological Diversity, Friends of the Earth (Washington, DC), and Defenders of Wildlife. Oil and gas interests included individual companies like BP, Chevron, and Shell, as well as industry trade associations, namely the American Petroleum Institute and the National Ocean Industries Association; government officials from Texas and Louisiana, including the Louisiana Attorney General, were also included and aligned with fossil fuel companies. In addition to the Biden White House, federal agencies that are responsible for energy development and endangered species protections were included, mainly NMFS, the Bureau of Ocean Energy Management, and the US Geological Survey. Federal judges who served on district and appellate courts that heard cases relevant to federal oil and gas lease sales in the Gulf region were also included, always in the form of quoting their rulings as it is not customary for judges to speak directly to reporters about specific cases in interviews or press conferences.

The focal threat to Rice’s whales in these texts was offshore oil and gas development, with some mention of ocean noise and vessel strikes as additional threats associated with energy exploration and drilling infrastructure. Two-thirds of these texts (*n* = 8) mentioned and cited the effects of the 2010 Deepwater Horizon oil spill as an event that killed at least 20% of the Rice’s whale population. In their quotes and background information provided to journalists, whale experts and advocates focused on the population estimates for Rice’s whales and scientific uncertainty to support their position of taking strong, precautionary measures on the part of the federal government to protect the world’s most endangered whale from industry activities to avoid extinction. In contrast, industry groups and their allies in state governments along the Gulf focused on the economics of oil and gas production in the region, legal requirements for the federal government to sell these leases, stronger industry safety standards as a result of the Deepwater Horizon oil spill, and the need to support the nation’s growing energy needs, using scientific uncertainty to argue that the costs of limiting oil and gas exploration in the area outweighed the benefits any protections to the Rice’s whale population, which they noted was a numerically small and geographically localized population.

### Evaluating the characteristics of Rice’s whale news coverage

Downs (1972) provides three characteristics of the public problems that are most likely to undergo an IAC. They are: (1) who the problem affects most, (2) the problem’s source, and (3) the problem’s excitement. We used the results from the CDA portion of this study to evaluate whether Rice’s whale SCP has any of these characteristics and determine if it is likely to undergo an IAC (Table 3). We conclude that this topic has all three characteristics and may undergo an IAC in the future.

**Table 3:** Characteristics of topics likely to undergo an IAC and examples from this analysis of news media about Rice’s whales

General characteristic	Definition	Examples from this case
*Who the problem affects most*	Most people in society do not suffer from the problem as much as some numerical or demographic minority	Few people directly interact with Rice’s whales or rely on them Extinction of the Rice’s whale would not likely affect some majority in the Gulf region or beyond unless it was associated with a broader ecological disaster or collapse
*The problem’s source*	The negative effects of the problem are the result of social arrangements that benefit to a majority group or a powerful minority in the population	Multiple human uses of Gulf waters include fossil fuel development, recreational and commercial fishing, recreational boating, and commercial shipping, all of which are economically and/or culturally important to the region, even if they pose threats to Rice’s whales Fossil fuels from the Gulf support the energy needs of the US and other industrialized nations, and are financially lucrative for corporations and their shareholders, even if they contribute to anthropogenic climate change that is a direct threat to Rice’s whale and marine ecosystems
*The problem’s excitement*	The problem has no intrinsically exciting qualities, or did and no longer does	Rice’s whales already existed and were protected, and are now classified under a new name Their biological, ecological, and behavioral traits are still being studied The existence of Rice’s whales is not inherently exciting until the social and governance dimensions are introduced and emphasized as a barrier to the species’ survival Research and policy change take place over longer time scales that can lose public interest

## Discussion

Using complementary CA and CDA methods to code and analyze 35 unique news media texts published between 2021 and 2024, the results of this study offer a preliminary understanding of an IAC focused on Rice’s whale SCP. Nineteen unique newspapers around the world published these texts, with more than half published by newspapers located in or near the US Gulf region. Journalist Tom McLaughlin, who writes for several Florida-based publications, was the leading author and produced nearly one-quarter of the analyzed texts (*n* = 8), equal to the amount published by all four of the other authors who published multiple articles in our sample. We found four key topics in the sampled coverage, which focused on (1) the research that led to the discovery of Rice’s whales as a distinct species, (2) attempts to exempt the US Air Force at Eglin Air Force Base from requirements of the ESA and MMPA, (3) an attempted vessel speed reduction and nighttime shipping ban to protect Rice’s whales, and (4) litigation related to oil and gas leasing in the Gulf. We also found that Rice’s whale SCP does appear to have the three characteristics of a topic likely to undergo an IAC in the future.

### Rice’s whale IAC stage

Based on our findings, we argue that this topic remains squarely in stage one of the IAC, the pre-problem stage, defined by the presence of some undesirable condition that mostly receives the attention of subject matter experts and the people most negatively affected (Downs 1972). With our data and findings, we argue that this topic is still one of regional rather than national or international interest, but not necessarily of importance. This is because we found only 35 unique texts published over four years, a majority of which were published by smaller news outlets located in the Gulf region and written by a small group of journalists, and there was not a clear catalyzing event that led policymakers and other human actor groups to commit to swift and decisive action in alignment with public interest in solving the problem. In fact, the discovery of Rice’s whales and attempts to strengthen conservation efforts through policy change met resistance and opposition from state and local policymakers, public administrators, citizens, and industry groups in the Gulf region. We conclude that the scientific confirmation and recognition of the Rice’s whale as a distinct and Critically Endangered species that is exclusive to the Gulf was not enough to advance the IAC, but instead creates a foundation for future advancement.

Remaining in the IAC’s pre-problem stage may not seem substantial at first, but there are important takeaways for scientists, professionals, and advocates who are working to support the recovery of the Rice’s whale population as they consider the role of communication and media in their current and future conservation, policy, and outreach efforts. While the Rice’s whale did receive representation in news media coverage, including in widely read national newspapers like *The New York Times* and *The Wall Street Journal*, the amount of coverage and where it is published are not defining indicators of an advancing IAC (Reamer and Rivera 2025). What matters most is *how* the topic is covered over time, though the two may be related. Downs (1972) defines the pre-problem stage of an IAC as coverage that centers on the people and organizations closest to the issue and who are most affected by it. In this case, those people were the stakeholders included in the sampled coverage: whale scientists, a coalition of environmental organizations led by the regional nonprofit, Healthy Gulf, Gulf area recreational fishers and boaters, fossil fuel companies, and the US Air Force at Eglin Air Force Base. Since the recognition of the Rice’s whale as a distinct species of only about 50 individuals—making it the world’s most endangered population of baleen whales—was not enough to advance the IAC, and it is unlikely that strategic communication campaigns on the part of science and environmental organizations would be enough to advance one without a catalyzing external event that puts the Rice’s whale population at further risk, our findings raise two important questions: what kind of event would advance the IAC and what can be done to prepare?

### Comparing the Rice’s whale and NARW IACs: Insights and recommendations

While scientists, conservation professionals, and environmental advocates had been researching, monitoring, rescuing, and advocating for stronger protections for NARWs for decades, it was not until the UME began in the summer of 2017 that the topic made its way into broader public view as part of an advancing IAC (Reamer et al. 2024). While the reporting that followed was aligned with scientific consensus related to the threats NARWs face and the conservation and policy solutions that could help the species recover, the nature of IACs is to simplify, dramatize, and problematize topics for readers to sustain their attention (Downs 1972; Reamer et al. 2024). What appears to be the dimension of greatest interest to news media organizations and their readers was the active and escalating conservation conflict between whale experts and advocates and lobster fishers and their allies as both sought to influence NMFS’ decision-making (Reamer et al. 2024; Reamer and Rivera 2025). The inflection point of the IAC followed the inflection point of the conservation conflict and has entered the fourth stage, gradual decline in intense public interest, now that the conflict has de-escalated (Reamer and Rivera 2025). With the NARW IAC past the point of peak public attention, policy action and organizational change is less likely and whale experts and advocates will need to consider this fact as they continue working to support the species’ recovery (Reamer and Rivera 2025). Since the NARW IAC is nearing the post-problem stage, we offer insights and recommendations about the role of news media in conservation that Rice’s whale scientists, conservationists, and advocates can use to prepare for their own possible IAC.

The Rice’s whale IAC has not yet advanced beyond the pre-problem stage and it is possible that the topic will remain in that stage for many years before it advances, if it ever does. This was the case for the NARW IAC from at least 2010 to 2017, even though the scientific consensus and ecological reality indicated the species needed stronger protections and public support sooner (Reamer et al. 2024). While the topic may remain a niche one for now, Rice’s whale experts and advocates do have time to be proactive and intentional about their use of communication and media—not just news media—to shape narratives and public perceptions to grow support for Rice’s whales and their conservation and management. Since IACs are difficult to predict and navigate, we recommend taking steps to continue tracking news media coverage about the topic and to develop evidence-based strategies that can be deployed if and when the IAC advances.

Based on Downs’ IAC (1972), previous findings about the NARW IAC, and our own analysis of news coverage about Rice’s whale SCP, we argue that a combination of an external event that puts the population of 50 whales at greater risk and a resulting conservation conflict as governments commit to policy change that would negatively affect human stakeholders in the region is the most likely scenario in which the IAC for this topic advances. If this were to be the case, it stands to reason that journalists and editors will emphasize conflict to pit conservation and industries in the Gulf directly at odds with one another to create an interesting story for their audiences. It appears that oil and gas development in the Gulf region would be the easiest dimension of the issue to simplify, dramatize, and problematize, followed by recreational fishing and boating, and possibly ocean noise created by military activities. This suggests that the kind of catalytic event that would advance the Rice’s whale IAC would be either an oil spill in the region, or a UME initiated by fishing gear entanglements , vessel strikes, and/or military activities in the region. Any of these events would be detrimental to the survival and recovery of the species and would put policymakers and industry groups in a reactive position rather than the proactive one that scientists and conservation groups have been advocating for.

Given the focus on conservation conflict during the second and third stages of the NARW IAC, we use Cusack et al.’s (2021) conservation conflict curve to identify the intensity of any conservation conflicts related to Rice’s whales based on the outcomes of our news media analysis. Across all four of the key topics we identified, we find the conservation conflicts surrounding Rice’s whales to be at level one of five. This stage, known as latent disagreement, is defined by an underlying conflict that is not apparent or visible and it is characterized by negative discourse among members of a stakeholder group about the interests of another stakeholder group (Cusack et al. 2021). We observed these latent disagreements and negative, but not always adversarial, discourses in the narratives surrounding Eglin Air Force Base activities, the petition for new vessel regulations, and oil and gas leases in the Gulf, mostly in coverage about the latter two topics. These negative discourses did not only originate with industry and special interest groups, but governmental organizations as well. Some human actor groups—like the Eglin Air Force Base—were generally supportive of actions to mitigate harm to the Rice’s whale population while others—like recreational fishing associations and oil and gas companies—were more dismissive of the need to take action. This is in contrast to the more adversarial and intense language used by stakeholder groups during the NARW IAC, especially lobster fishers and their allies in business and government who spoke of the certain economic and social harms that would result from any new or stronger fishing regulations designed to protect NARWs from fishing gear entanglement, and in pointing to commercial shipping, cruise lines, and Canadian lobster fishing as what they believed to be the true threats to NARWs (Reamer et al. 2024). If the conflict in any one or more of these dimensions of Rice’s whale conservation and management were to escalate along Cusack et al.’s (2021) conservation conflict curve, it is likely that the IAC would advance due to the presence of more elements that journalists and editors could use to simplify, dramatize, and problematize the issue for their audiences. Increased attention in this form would not necessarily be beneficial to Rice’s whale conservation and management as it could create opportunities for other actor groups to use communication and media in support of their interests and change existing power dynamics and public opinion (Nisbet and Huge 2007), which the New England lobster fishing industry was able to do during stages two and three of the NARW IAC (Reamer et al. 2024).

Where NARW SCP had the support of state and local governments along its migratory range (Reamer et al. 2024)—even if many of those governments expressed equal support for lobster fishing interests—our data show a degree of opposition to Rice’s whale conservation and policy from state and local governments in the Gulf region, representing both the executive and legislative branches. This opposition appears to be rooted in a tension between public values (Boyle 2001), with state and local governments featured in the sampled coverage about recreational fishing and boating as well as oil and gas development favoring economic development and prosperity and personal freedoms above nearly all else, citing the importance of these activities to their local culture and economies. Another notable difference between the two whale IACs is that reporting about the NARW presented anthropogenic climate change as a more passive, tertiary threat to the population (Reamer et al. 2024) that makes the leading threats of fishing gear entanglements and vessel strikes worse as the whales follow shifting prey sources, whereas reporting about Rice’s whales mentions it as a source of several leading threats, directly tying fossil fuel development in the Gulf region to anthropogenic climate change. Rice’s whale SCP may face a challenging political environment as the US federal government pursues increased fossil fuel production in the Gulf and attempts to weaken environmental laws in favor of economic interests, which may further polarize stakeholder groups in ways that media may take notice of or emphasize, adding additional challenges to conservation and management efforts.

If the external event that leads to an advancing IAC is an oil spill or an associated event related to fossil fuel development in the Gulf, it is possible that oil and gas companies will leverage scientific uncertainty, journalistic norms, and deploy sophisticated misinformation and disinformation campaigns to sow doubt, shift blame, and sway public opinion and policy decisions as they have done with other contexts in the past (Oreskes and Supran 2017; Lamb et al. 2020; Goldberg and Vandenberg 2021; Kaupa 2021; Maani et al. 2022). While it may be easy for public opinion to side with whale conservationists in such a hypothetical scenario, it would likely be too late as an oil spill event would have devastating and permanent consequences for the already small Rice’s whale population and their ecosystem. Continued research and tracking of media about Rice’s whale SCP may help identify such tactics and strategies early and support the development of counter messaging. We also recommend that scientists, professionals, and advocates continue using communication and media to reach non-expert audiences and policymakers about this Critically Endangered whale species and its conservation and to support investments in related research to support the development of evidence-based communication strategies as Rice's whale conservation and management evolves alongside changes in the social, political, and ecological environments .

To that end,  we recommend continuing to track the IAC through news media coverage on at least a biennial basis to help identify if and when the topic might advance and any conservation conflicts may escalate, which relevant organizations, communicators, and advocates could use to develop more adaptable and effective communication and outreach strategies. To develop a deeper understanding of how Rice’s whale SCP is being represented in media, we also recommend research that goes beyond news media by exploring a broader range of past, current, and future media like documentary films, nonprofit advocacy campaigns, and social media focused on Rice’s whale SCP. We also recommend research that measures a baseline of public knowledge, attitudes, and beliefs about Rice’s whales in Gulf area communities while the topic remains in stage 1 of the IAC and, thus, of greatest interest regionally; the outcomes may help define audience segments and triangulate where communication and outreach efforts are either most needed or could be most effective. We also recommend research that measures policy support at the local, state, and federal levels, and engaging with stakeholder groups that are closest to the issue, like environmental groups and trade associations, to better understand the social and governance challenges and opportunities associated with Rice’s whale conservation and management under current and future conditions. Experimental studies testing the effectiveness of specific communication interventions related to Rice’s whale SCP—such as message framings, identifying trusted messengers, or different media formats—may also be valuable. These efforts and others can all provide the empirical evidence that environmental organizations and communicators often lack during planning stages (Liang et al. 2018; Waldo et al. 2025). Such resources  can support evidence-based decision-making as communicators and advocates develop and implement strategies that shape narratives, reach new audiences and broaden public knowledge of the species and cause, and, ultimately, increase public and policymaker support for Rice’s whales and their conservation. Funders can be supportive by including strategic communication and outreach in their funding for conservation research and practice or offering funds specifically for these essential activities.

### Informed insights and future directions for other conservation and sustainability topics

Human communication and media are essential to SES and comprehensive strategies for conservation and social change (Brüggemann et al. 2023). As we have shown, science and media organizations operate from different logics, on different timelines, and in pursuit of different goals, making the study and application of strategic environmental communication best practices worthwhile investments (Liang et al. 2018). While this case focuses on Rice’s whale SCP in news media, it offers an approach and lessons learned that scholars, professionals, and advocates focused on other conservation topics in other geographies can use to support their own work.

Studying human communication and media can offer valuable insights to help understand and navigate conservation scenarios, whether by listening for stakeholder values, worldviews, and beliefs, or areas of difference and opportunity (Staddon et al. 2023; Blackett et al. 2024), or by identifying effective strategies, tactics, mediums, and messages to influence public opinion, discourse, and policy decisions (Liang et al. 2018). Our use of CA and CDA to explore IACs is just one way to collect and analyze data from media to produce actionable knowledge. Investing in interdisciplinary approaches to conservation research can enrich understanding of the roles media producers and consumers, and media itself play in SES  and conservation practice. Following the three-stage model of media—studying media production, their contents, and audience effects—can offer a more holistic view of human communication and media in conservation (Painter 2021). Moreover, the analytical approach used in our study can be adapted by communications and external affairs teams with environmental organizations to move beyond media monitoring that tracks mentions and other surface-level metrics and toward strategic content analysis that informs outreach, advocacy, and policy engagement. External affair teams representing environmental organizations can use the findings of those efforts to help teams across their organizations understand at a deeper level the environmental, social, and governance dimensions of the topics they consider a priority, and identify how peer and opposing groups are using communication and media to shape public discourses. We also recommend continued research on IACs centered around other  conservation topics, which may allow for more comprehensive analysis to create a deeper understanding of media systems in conservation and effective strategies and tools to use to support conservation goals. For example, with more IAC case studies, there may be opportunities for theoretical research that investigates and measures relationships between escalating conservation conflicts using a framework like Cusack et al.’s (2021) conservation conflict curve and IACs about conservation topics.

In terms of practical applications, we echo recommendations to make continued investments in relationship building and training for scientists and journalism professionals to improve environmental reporting, especially for marine and coastal topics (Kolandai-Matchett et al. 2021). We also recommend continued investment by environmental organizations in their communications teams or partnerships with researchers to conduct social listening work to understand how stakeholder groups and media producers are using language and media to shape public perceptions, discourses, and decision-making about the topics they consider a priority. Finally, we recommend academic programs at the undergraduate and graduate levels—and organizations that offer professional development for organizations and conservation professionals—integrate environmental communication and media studies into their core curricula to train boundary-spanning conservation professionals who can navigate complex media systems in support of making environmental science accessible and actionable (Arnott et al. 2020; Goodrich et al. 2020; Mach et al. 2020).

### Limitations

This study has several limitations. This work is exploratory in its nature and may not represent the full range of texts about the topic of interest. Additionally, the analysis was limited to newspaper articles and did not include other influential media formats such as cable news, radio, public broadcasting (e.g., NPR, PBS), or podcasts. All of the texts we analyzed were published in English, which may have been a bias in the database that provided, but it may also show that outlets and readers in Mexico and other nations proximal to the Rice’s whale’s habitat may not have taken significant interest in reporting on the subject. With a focus on the contents of media, this  study does not explore the motivations behind journalists’ source selection, the dynamics of choosing and engaging with sources for quotes and background information, or the co-creation of media reporting. Similarly, this study does not consider the reach or readership of the texts we analyzed, nor does it offer insights about the effects of media coverage on audiences in the form of public discussion, opinion, or policy outcomes. And, finally, we took steps to ensure reliability and minimize biases, but bias in coding and analysis cannot ever be fully eliminated in research of this nature.

## Conclusion

In this study, we examined how Rice’s whale science, conservation, and policy were represented in news media between 2021 and 2024. We used content and critical discourse analysis to analyze 35 articles from 19 newspapers. We found that the issue remains in the pre-problem stage of the issue-attention cycle, with coverage that was mostly regional  and focused on a narrow set of stakeholders and topics. Despite the species’ Critically Endangered status and its title of the world's most endangered great whale, media attention has not yet catalyzed widespread public or policy engagement in support of the Rice's whale's conservation and management. However, the presence of latent conservation conflict and the potential for catalytic events that could harm the Rice's whale population suggest that the issue could escalate in visibility and urgency in the future. By understanding the dynamics of media representation and stakeholder discourse, conservation scientists, professionals, and advocates can better prepare for future emergence of an issue-attention cycle by continuing to use strategic communication and media to bring attention to the Rice's whale and its conservation, and develop communication strategies that are both proactive and evidence-based. These insights are not only relevant for Rice’s whale conservation as our methods are transferable and the outcomes of this kind of work can helpunderstand and navigate media systems in other conservation contexts.

## Data Availability

Due to copyright restrictions the data and necessary documents cannot be publicly posted. The data and documents supporting this research will be made available upon reasonable request of the corresponding author.

## References

[CR1] Arnott, J. C., K. J. Mach, and G. Wong-Parodi. 2020. The science of actionable knowledge. *Current Opinion in Environmental Sustainability* 42: A1–A5. 10.1016/j.cosust.2020.03.007.

[CR2] Bailey, I. 2022. Media coverage, attention cycles and the governance of plastics pollution. *Environmental Policy and Governance* 32: 377–389. 10.1002/eet.1977.

[CR3] Baumeister, R. F., E. Bratslavsky, C. Finkenauer, and K. D. Vohs. 2001. Bad is Stronger than Good. *Review of General Psychology* 5: 323–370. 10.1037/1089-2680.5.4.323.

[CR4] Bebbington, K., C. MacLeod, T. M. Ellison, and N. Fay. 2017. The sky is falling: Evidence of a negativity bias in the social transmission of information. *Evolution and Human Behavior* 38: 92–101. 10.1016/j.evolhumbehav.2016.07.004.

[CR5] Bejder, M., D. W. Johnston, J. Smith, A. Friedlaender, and L. Bejder. 2016. Embracing conservation success of recovering humpback whale populations: Evaluating the case for downlisting their conservation status in Australia. *Marine Policy* 66: 137–141. 10.1016/j.marpol.2015.05.007.

[CR6] Bestor, C. 2023. A great win for Destin’s fishing industry”: Okaloosa commissioners react to NOAA rejecting rules on Rice’s whales. *Pensacola News Journal*, A4.

[CR7] Blackett, P., E. Le Heron, S. Awatere, R. Le Heron, J. Logie, J. Hyslop, J. Ellis, F. Stephenson, et al. 2024. Navigating Choppy Waters: Why are we always arguing about risk and uncertainty in marine multi-use environments and what can we do about it? *Policy Quarterly* 20: 62–68. 10.26686/pq.v20i3.9560.

[CR8] Boan, J. J., J. R. Malcolm, M. D. Vanier, D. L. Euler, and F. M. Moola. 2018. From climate to caribou: How manufactured uncertainty is affecting wildlife management. *Wildlife Society Bulletin* 42: 366–381. 10.1002/wsb.891.

[CR9] Bossart, G. D. 2011. Marine Mammals as Sentinel Species for Oceans and Human Health. *Veterinary Pathology* 48: 676–690. 10.1177/0300985810388525. 21160025 10.1177/0300985810388525

[CR10] Boykoff, M. T. 2009. We Speak for the Trees: Media Reporting on the Environment. *Annual Review of Environment and Resources* 34: 431–457. 10.1146/annurev.environ.051308.084254.

[CR11] Boyle, P. 2001. Public problems, values, and choices. *Popular Government* 1: 18–23.

[CR12] Brito, C., N. Vieira, and J. G. Freitas. 2019. The wonder whale: A commodity, a monster, a show and an icon. *Anthropozoologica* 54: 13. 10.5252/anthropozoologica2019v54a3.

[CR13] Brüggemann, M., A. Carvalho, B. Brevini, and J. Downe. 2023. Still Watching from the Sidelines? The Case for Transformative Environmental Communication Scholarship. *International Journal of Communication* 17: 5039–5052.

[CR14] Carmi, N., and S. Kimhi. 2015. Further Than the Eye Can See: Psychological Distance and Perception of Environmental Threats. *Human and Ecological Risk Assessment: An International Journal* 21: 2239–2257. 10.1080/10807039.2015.1046419.

[CR15] Charmaz, K. 1995. Grounded Theory. In *Rethinking Methods in Psychology*, ed. J. Smith, R. Harré, and L. Langenhove, 27–49. SAGE Publications Ltd. 10.4135/9781446221792.n3.

[CR16] Clapham, P. J. 2016. Managing Leviathan: Conservation Challenges for the Great Whales in a Post-Whaling World. *Oceanography* 29: 214–225. 10.5670/oceanog.2016.70.

[CR17] Conrad, J. 1987. The issue-attention cycle: A question of impact on energy conservation policy in West Germany. *Energy Policy* 15: 567–570. 10.1016/0301-4215(87)90168-6.

[CR18] Cook, J. 2022. Understanding and Countering Misinformation About Climate Change: In I. R. Management Association (Ed.), *Research Anthology on Environmental and Societal Impacts of Climate Change* (pp. 1633–1658). IGI Global. 10.4018/978-1-6684-3686-8.ch081

[CR19] Corkeron, P., Reeves, R., and Rosel, P. 2017. *Balaenoptera edeni (Gulf of Mexico subpopulation). The IUCN Red List of Threatened Species.* (e. T117636167A117636174) . The IUCN Red List of Threatened Species 2017. 10.2305/IUCN.UK.2017-3.RLTS.T117636167A117636174.en

[CR20] Cusack, J. J., T. Bradfer-Lawrence, Z. Baynham-Herd, Y. Castelló, S. Tickell, I. Duporge, H. Hegre, L. Moreno Zárate, et al. 2021. Measuring the intensity of conflicts in conservation. *Conservation Letters* 14: e12783. 10.1111/conl.12783. 34434253 10.1111/conl.12783 PMC8365684

[CR21] Djerf-Pierre, M. 2013. Green metacycles of attention: Reassessing the attention cycles of environmental news reporting 1961–2010. *Public Understanding of Science* 22: 495–512. 10.1177/0963662511426819. 23833112 10.1177/0963662511426819

[CR22] Downing, J. A. 2024. What’s hot and what’s not in the aquatic sciences—Understanding and improving news coverage. *Limnology and Oceanography Letters* 9: 674–682. 10.1002/lol2.10425.

[CR23] Downs, A. 1972. Up and Down with Ecology—The “issue-attention” cycle. *The Public Interest* 28: 38–50.

[CR24] Editorial Board. 2023. Biden’s Summer Regulatory Onslaught. *The Wall Street Journal*, A16.

[CR25] Farber, D. A. 2015. Separated at Birth? Addressing the Twin Global Crises of Biodiversity and Climate Change. *SSRN Electronic Journal*. 10.2139/ssrn.2593498.

[CR26] Garrison, E. G., G. S. Morgan, K. McGrath, C. Speller, and A. Cherkinsky. 2019. Recent dating of extinct Atlantic gray whale fossils, *(Eschrichtius robustus),* Georgia Bight and Florida, western Atlantic Ocean. *PeerJ* 7: e6381. 10.7717/peerj.6381. 30746309 10.7717/peerj.6381 PMC6368218

[CR27] Garrison, L., M. Soldevilla, A. Martinez, and K. Mullin. 2024. A density surface model describing the habitat of the Critically Endangered Rice’s whale Balaenoptera ricei in the Gulf of Mexico. *Endangered Species Research* 54: 41–58. 10.3354/esr01324.

[CR28] Geschke, J., M. C. Rillig, K. Böhning-Gaese, T. Potthast, A. Arth, L. V. Dicks, F. Habekuss, D. Kleinschmit, et al. 2023. Science journalism and a multi-directional science-policy-society dialogue are needed to foster public awareness for biodiversity and its conservation. *PLOS Sustainability and Transformation* 2: e0000083. 10.1371/journal.pstr.0000083.

[CR29] Giovos, I., D. K. Moutopoulos, S. Nakagun, N. Vieira, E. Akritopoulou, A. Floriou-Servou, B. Savinelli, M. Papadopoulos, et al. 2019. An International Online Social Survey of Public Attitudes Towards Cetaceans. *Aquatic Mammals* 45: 327–339. 10.1578/AM.45.3.2019.327.

[CR30] Goldberg, R. F., and L. N. Vandenberg. 2021. The science of spin: Targeted strategies to manufacture doubt with detrimental effects on environmental and public health. *Environmental Health* 20: 33. 10.1186/s12940-021-00723-0. 33771171 10.1186/s12940-021-00723-0 PMC7996119

[CR31] Goodrich, K. A., K. D. Sjostrom, C. Vaughan, L. Nichols, A. Bednarek, and M. C. Lemos. 2020. Who are boundary spanners and how can we support them in making knowledge more actionable in sustainability fields? *Current Opinion in Environmental Sustainability* 42: 45–51. 10.1016/j.cosust.2020.01.001.

[CR32] Hardy, C., B. Harley, and N. Phillips. 2004. Discourse Analysis and Content Analysis: Two Solitudes? *Qualitative Methods* 2: 19–22. 10.5281/zenodo.998649

[CR33] Harrison, H. L., and P. A. Loring. 2020. Seeing beneath disputes: A transdisciplinary framework for diagnosing complex conservation conflicts. *Biological Conservation* 248: 108670. 10.1016/j.biocon.2020.108670.

[CR34] Hayes, S. A., E. Josephson, K. Maze-Foley, P. E., Rosel, B. Byrd, S. Chavez-Rosales, T. V. N. Cole, L. P. Garrison, et al. 2020. *U.S. Atlantic and Gulf of Mexico marine mammal stock assessments—2019* (p. 468) [Technical Memorandum]. Northeast Fisheries Science Center (U.S.). 10.25923/ngsq-qc69

[CR35] Hilgartner, S., and C. L. Bosk. 1988. The Rise and Fall of Social Problems: A Public Arenas Model. *The American Journal of Sociology* 94: 53–78. 10.1086/228951.

[CR36] Hodgson, I. D., A. Fischer, S. M. Redpath, and J. C. Young. 2022. Fight or Flight? Understanding Different Stakeholder Responses to Conservation Conflicts. *Society & Natural Resources* 35: 628–645. 10.1080/08941920.2022.2048933.

[CR37] Holtzhausen, D. R., and A. Zerfass, eds. 2015. *The Routledge handbook of strategic communication*. Routledge.

[CR38] Hoyt, E., and E. C. M. Parsons. 2014. The whale-watching industry: Historical development. In *whale-watching*, 1st ed., ed. J. Higham, L. Bejder, and R. Williams, 57–70. Cambridge University Press. 10.1017/CBO9781139018166.006.

[CR40] Jahoda, M., M. Zanardelli, F. Dhermain, J. Alessi, F. Armonio, M. Ballardini, A. Barcelo, G. Calogero, et al. 2025. Codamozza-Fluker: The Compelling Case of a Flukeless Fin Whale Traveling Throughout the Mediterranean Sea and the Need for Basin-Wide Conservation Efforts. *Ecology and Evolution* 15: e71313. 10.1002/ece3.71313. 40406589 10.1002/ece3.71313 PMC12094965

[CR39] Jefferson, T. A., and A. J. Schiro. 1997. Distribution of cetaceans in the offshore Gulf of Mexico. *Mammal Review* 27: 27–50. 10.1111/j.1365-2907.1997.tb00371.x.

[CR41] Kalland, A. 2009. *Unveiling the Whale: Discourses on Whales and Whaling*. Berghahn Books. 10.2307/j.ctt9qd9tk.

[CR42] Kaupa, C. 2021. Smoke Gets in Your Eyes: Misleading Fossil Fuel Advertisement in the Climate Crisis. *Journal of European Consumer and Market Law* 10: 21–30. 10.2139/ssrn.3786647

[CR43] Kellert, S. R. 1985. American Attitudes Toward and Knowledge of Animals: An Update. In *Advances in Animal Welfare Science 1984*, ed. M. W. Fox and L. D. Mickley, 177–213. Netherlands: Springer. 10.1007/978-94-009-4998-0_11.

[CR44] Kolandai-Matchett, K., and M. Armoudian. 2020. Message framing strategies for effective marine conservation communication. *Aquatic Conservation: Marine and Freshwater Ecosystems* 30: 2441–2463. 10.1002/aqc.3349.

[CR45] Kolandai-Matchett, K., M. Armoudian, S. Thrush, J. Hillman, L. Schwendenmann, J. Jakobsson, T. Haggitt, C. O’Hara Blain, et al. 2021. Marine ecosystem science and the media: Exploring ways to improve news coverage through journalist–scientist working relations. *Aquatic Conservation: Marine and Freshwater Ecosystems* 31: 3034–3055. 10.1002/aqc.3708.

[CR46] Kraus, S. D., M. W. Brown, H. Caswell, C. W. Clark, M. Fujiwara, P. K. Hamilton, R. D. Kenney, A. R. Knowlton, et al. 2005. North Atlantic Right Whales in Crisis. *Science* 309: 561–562. 10.1126/science.1111200. 16040692 10.1126/science.1111200

[CR47] Lamb, W. F., G. Mattioli, S. Levi, J. T. Roberts, S. Capstick, F. Creutzig, J. C. Minx, F. Müller-Hansen, et al. 2020. Discourses of climate delay. *Global Sustainability* 3: e17. 10.1017/sus.2020.13.

[CR48] Learn, J. R. 2021. A “uniquely American whale”: New species discovered off southern US coast. *The Guardian*. https://www.theguardian.com/environment/2021/feb/08/rices-whales-new-species-discovered-southern-us-coast

[CR49] Legagneux, P., N. Casajus, K. Cazelles, C. Chevallier, M. Chevrinais, L. Guéry, C. Jacquet, M. Jaffré, et al. 2018. Our House Is Burning: Discrepancy in Climate Change vs. Biodiversity Coverage in the Media as Compared to Scientific Literature. *Frontiers in Ecology and Evolution* 5: 175. 10.3389/fevo.2017.00175.

[CR50] Liang, Y., K. F. Kee, and L. K. Henderson. 2018. Towards an integrated model of strategic environmental communication: Advancing theories of reactance and planned behavior in a water conservation context. *Journal of Applied Communication Research* 46: 135–154. 10.1080/00909882.2018.1437924.

[CR51] Lindquist, O. 2000. *The North Atlantic gray whale (Escherichtius robustus): An historical outline based on Icelandic, Danish-Icelandic, English and Swedish sources dating from ca 1000 AD to 1792*. Andrews and Stirling, Scotland: Universities of St.

[CR52] Lörcher, I., and I. Neverla. 2015. The Dynamics of Issue Attention in Online Communication on Climate Change. *Media and Communication* 3: 17–33. 10.17645/mac.v3i1.253.

[CR53] Lupia, A. 2013. Communicating science in politicized environments. *Proceedings of the National Academy of Sciences* 110: 14048–14054. 10.1073/pnas.1212726110. 10.1073/pnas.1212726110 PMC3752174 23940336

[CR54] Maani, N., M. C. I. Van Schalkwyk, F. T. Filippidis, C. Knai, and M. Petticrew. 2022. Manufacturing doubt: Assessing the effects of independent vs industry-sponsored messaging about the harms of fossil fuels, smoking, alcohol, and sugar sweetened beverages. *SSM - Population Health* 17: 101009. 10.1016/j.ssmph.2021.101009. 35036514 10.1016/j.ssmph.2021.101009 PMC8749266

[CR55] Mach, K. J., M. C. Lemos, A. M. Meadow, C. Wyborn, N. Klenk, J. C. Arnott, N. M. Ardoin, C. Fieseler, et al. 2020. Actionable knowledge and the art of engagement. *Current Opinion in Environmental Sustainability* 42: 30–37. 10.1016/j.cosust.2020.01.002.

[CR56] Mast, R., D. N. Castelblanco-Martínez, and A. Hemphill. 2014. Sea Mammals. In *Handbook of the Mammals of the World*, vol. 4, ed. D. E. Wilson and R. A. Mittermeier, 17–32. Lynx Nature Books.

[CR105] Mazzoldi, C., G. Bearzi, C. Brito, I. Carvalho, E. Desiderà, L. Endrizzi, L. Freitas, E. Giacomello, et al. 2019. From sea monsters to charismatic megafauna: Changes in perception and use of large marine animals. *PLOS ONE* 14 : e0226810. 10.1371/journal.pone.0226810. 10.1371/journal.pone.0226810 PMC6938407 31891944

[CR57] McComas, K., and J. Shanahan. 1999. Telling Stories About Global Climate Change: Measuring the Impact of Narratives on Issue Cycles. *Communication Research* 26: 30–57. 10.1177/009365099026001003.

[CR58] McCombs, M. E., and D. L. Shaw. 1972. The Agenda-Setting Function of Mass Media. *Public Opinion Quarterly* 36: 176–187. 10.1086/267990.

[CR59] McLaughlin, T. 2022. *Newly discovered Gulf whale species teeters on the brink of extinction*, 5. The News Press.

[CR60] McLaughlin, T. 2023. Rep. Gaetz seeks to strip protections from Rice’s Whale. *Pensacola News Journal*, A1.

[CR61] McLaughlin, T. 2024a. Rep. Gaetz seeks to force Eglin Air Force Base to halt protections of Rice’s Whale. *Pensacola News Journal*, A1.

[CR62] McLaughlin, T. 2024b. Gaetz continues to push for Gulf test range. *Pensacola News Journal*, A3.

[CR63] McLuhan, M. 1964. *Understanding Media: The Extensions of Man*, 1st ed. McGraw-Hill.

[CR64] Merkley, E. 2020. Are Experts (News)Worthy? Balance, Conflict, and Mass Media Coverage of Expert Consensus. *Political Communication* 37: 530–549. 10.1080/10584609.2020.1713269.

[CR65] Naylor, W., and E.C.M. Parsons. 2018. An Online Survey of Public Knowledge, Attitudes, and Perceptions Toward Whales and Dolphins, and Their Conservation. *Frontiers in Marine Science* 5: 1–17. 10.3389/fmars.2018.00153.29552559

[CR66] Nelms, S., J. Alfaro-Shigueto, J. Arnould, I. Avila, S. Bengtson Nash, E. Campbell, M. Carter, T. Collins, et al. 2021. Marine mammal conservation: Over the horizon. *Endangered Species Research* 44: 291–325. 10.3354/esr01115.

[CR67] Neuman, W., L. Guggenheim, S. Mo Jang, and S. Y. Bae. 2014. The Dynamics of Public Attention: Agenda-Setting Theory Meets Big Data: Dynamics of Public Attention. *Journal of Communication* 64: 193–214. 10.1111/jcom.12088.

[CR68] Nisbet, M. C., and Huge, M. 2007. *Where do science debates come from? Understanding attention cycles and framing *(pp. 193–230). CABI. 10.1079/9781845932046.0193

[CR69] NOAA Fisheries. 2025. *Rice’s Whale | NOAA Fisheries* (Southeast). NOAA. https://www.fisheries.noaa.gov/species/rices-whale

[CR70] Noad, M. J., E. Kniest, and R. A. Dunlop. 2019. Boom to bust? Implications for the continued rapid growth of the eastern Australian humpback whale population despite recovery. *Population Ecology* 61: 198–209. 10.1002/1438-390X.1014.

[CR71] Painter, J. 2021. The International Coverage of Biodiversity Loss. In *The Handbook of International Trends in Environmental Communication*, 1st ed., ed. B. Takahashi, J. Metag, J. Thaker, and S. E. Comfort, 173–189. Routledge. 10.4324/9780367275204-13.

[CR72] Petersen, K.K. 2009. Revisiting Downs’ Issue-Attention Cycle: International Terrorism and U.S. Public Opinion. *Journal of Strategic Security* 2: 1–16. 10.5038/1944-0472.2.4.1.

[CR73] Peterson, M. J. 1992. Whalers, cetologists, environmentalists, and the international management of whaling. *International Organization* 46: 147–186. 10.1017/S0020818300001478.

[CR74] Pettis, H. M., and P. K. Hamilton. 2024. North Atlantic Right Whale Consortium 2023 Report Card. *North Atlantic Right Whale Consortium*. 10.1575/1912/69694.

[CR75] Pinto, B., and A. Matias. 2023. European journalists and the sea: Contexts, motivations, and difficulties. *Public Understanding of Science* 32: 459–469. 10.1177/09636625221137036. 36448498 10.1177/09636625221137036 PMC10115933

[CR76] Putnam, L.L., and M. Shoemaker. 2007. Changes in conflict framing in the news coverage of an environmental conflict. *Journal of Dispute Resolution* 2007: 167. Retrieved from: https://scholarship.law.missouri.edu/jdr/vol2007/iss1/10

[CR77] Reamer, M. 2024. *Environmental Media and the Conservation and Management of the Critically Endangered North Atlantic Right Whale* [Dissertation, University of Miami]. https://scholarship.miami.edu/esploro/outputs/doctoral/Environmental-Media-and-the-Conservation-and/991032490271502976?institution=01UOML_INST#metrics

[CR78] Reamer, M. B. 2022a. Communicating ocean and human health connections: An agenda for research and practice. *Frontiers in Public Health* 10: 1033905. 10.3389/fpubh.2022.1033905. 36530715 10.3389/fpubh.2022.1033905 PMC9755358

[CR79] Reamer, M. B. 2022b. Recovery of the Eastern North Pacific Gray Whale: A Case Study. *Journal of International Wildlife Law & Policy* 25: 201–240. 10.1080/13880292.2022.2146850.

[CR80] Reamer, M., H. Vaughan, and M. Shriver-Rice. 2023. Documenting the Endangered North Atlantic Right Whale: Policies, Conservation Efforts, and Stakeholders Depicted in *Entangled* and *Last of the Right Whales*. *Journal of International Wildlife Law & Policy* 26: 281–306. 10.1080/13880292.2023.2294590.

[CR81] Reamer, M., C. Macdonald, J. Wester, R. Fielding, and M. Shriver-Rice. 2024. A “war” over lobster and whales: The issue-attention cycle, media discourse, and political ecology of right whale science and conservation in six US newspapers. *Frontiers in Communication* 9: 1417414. 10.3389/fcomm.2024.1417414.

[CR82] Reamer, M., and E. Rivera. 2025. Following the issue-attention cycle of North Atlantic right whale science, conservation, and policy in six US newspapers: 2023 and 2024. *Environmental Research Communications* 7 : 71006. 10.1088/2515-7620/adeeec.

[CR83] Reiss, D., J. Sickler, S. Gruber, P. Boyle, E. Elliott, K. Lemcke, J. Fraser, and B. Newman. 2006. Dolphins in Popular Literature and Media. *Society & Animals* 14: 321–349. 10.1163/156853006778882402.

[CR106] Reynolds III, J., H. Marsh, and T. Ragen. 2009. Marine Mammal Conservation. *Endangered Species Research* 7: 23–28. 10.3354/esr00179.

[CR84] Rice, D. 1965. Bryde’s whale in the Gulf of Mexico. *Norsk Hvalfangst-Tidende* 54: 114–115.

[CR85] Rosel, P., Corkeron, P., & Soldevilla, M. 2022. *Balaenoptera ricei:The IUCN Red List of Threatened Species* (e. T215823373A208496244) . 10.2305/IUCN.UK.2022-1.RLTS.T215823373A208496244.en

[CR86] Rosel, P. E., L. A. Wilcox, T. K. Yamada, and K. D. Mullin. 2021. A new species of baleen whale ( *Balaenoptera* ) from the Gulf of Mexico, with a review of its geographic distribution. *Marine Mammal Science* 37: 577–610. 10.1111/mms.12776.

[CR87] Sachsman, D. B., and J. M. Valenti, eds. 2020. *Routledge Handbook of Environmental Journalism*. Roultledge, Taylor & Francis Group.

[CR88] Schuldt, J. P., K. A. McComas, and S. E. Byrne. 2016. Communicating about ocean health: Theoretical and practical considerations. *Philosophical Transactions of the Royal Society B: Biological Sciences* 371: 20150214. 10.1098/rstb.2015.0214. 10.1098/rstb.2015.0214 PMC4760144 26880833

[CR89] Scott, N. J., and E. C. M. Parsons. 2005. A survey of public opinion in south-west Scotland on cetacean conservation issues. *Aquatic Conservation: Marine and Freshwater Ecosystems* 15: 299–312. 10.1002/aqc.662.

[CR90] Shoemaker, P. J., and S. D. Reese. 2014. *Mediating the message in the 21st century: A media sociology perspective*, 3rd ed. Routledge, Taylor & Francis Group.

[CR91] Soldevilla, M., A. Debich, L. Garrison, J. Hildebrand, and S. Wiggins. 2022. Rice’s whales in the northwestern Gulf of Mexico: Call variation and occurrence beyond the known core habitat. *Endangered Species Research* 48: 155–174. 10.3354/esr01196.

[CR92] Soldevilla, M. S., A. J. Debich, I. Pérez-Carballo, S. Jarriel, K. E. Frasier, L. P. Garrison, A. Gracia, J. A. Hildebrand, et al. 2024. Rice’s whale occurrence in the western Gulf of Mexico from passive acoustic recordings. *Marine Mammal Science* 40: e13109. 10.1111/mms.13109.

[CR93] Soroka, S., P. Fournier, and L. Nir. 2019. Cross-national evidence of a negativity bias in psychophysiological reactions to news. *Proceedings of the National Academy of Sciences* 116: 18888–18892. 10.1073/pnas.1908369116. 10.1073/pnas.1908369116 PMC6754543 31481621

[CR94] Staddon, S., A. Byg, M. Chapman, R. Fish, A. Hague, and K. Horgan. 2023. The value of listening and listening for values in conservation. *People and Nature* 5: 343–356. 10.1002/pan3.10232.

[CR95] Supran, G., and N. Oreskes. 2017. Assessing ExxonMobil’s climate change communications (1977–2014). *Environmental Research Letters* 12: 084019. 10.1088/1748-9326/aa815f.

[CR96] Takahashi, B., and E. C. Tandoc. 2016. Media sources, credibility, and perceptions of science: Learning about how people learn about science. *Public Understanding of Science* 25: 674–690. 10.1177/0963662515574986. 25792288 10.1177/0963662515574986

[CR97] Tang, Y.-T., and W.-T. Chooi. 2023. From concern to action: The role of psychological distance in attitude towards environmental issues. *Current Psychology* 42: 26570–26586. 10.1007/s12144-022-03774-9.

[CR98] Thompson-Saud, G., S. Gelcich, and J. Barraza. 2018. Marine environmental issues in the mass media: Insights from television, newspaper and internet searches in Chile. *Ocean & Coastal Management* 165: 154–160. 10.1016/j.ocecoaman.2018.08.015.

[CR99] Tiller, R., F. Arenas, C. Galdies, F. Leitão, A. Malej, B. M. Romera, C. Solidoro, R. Stojanov, et al. 2019. Who cares about ocean acidification in the Plasticene? *Ocean & Coastal Management* 174: 170–180. 10.1016/j.ocecoaman.2019.03.020.

[CR100] Veríssimo, D., D. C. MacMillan, R. J. Smith, J. Crees, and Z. G. Davies. 2014. Has Climate Change Taken Prominence over Biodiversity Conservation? *BioScience* 64: 625–629. 10.1093/biosci/biu079.

[CR101] Vincent, A. C. J. 2011. Saving the shallows: Focusing marine conservation where people might care. *Aquatic Conservation: Marine and Freshwater Ecosystems* 21: 495–499. 10.1002/aqc.1226.

[CR102] Waldo, J. L., M. D. Needham, and M. S. Jones. 2025. How can we sea change? Audience subgroups and psychological cognitions to target in action-oriented ocean change communication. *Marine Policy* 173: 106585. 10.1016/j.marpol.2024.106585.

[CR103] Wilcox Talbot, L. A., N. L. Vollmer, A. Martinez, L. A. Dias, L. P. Garrison, and P. E. Rosel. 2025. Validated Environmental DNA Assay for Detection of the Rare Rice’s Whale (Balaenoptera ricei). *Environmental DNA* 7: e70074. 10.1002/edn3.70074.

[CR104] Zerbini, A. N., G. Adams, J. Best, P. J. Clapham, J. A. Jackson, and A. E. Punt. 2019. Assessing the recovery of an Antarctic predator from historical exploitation. *Royal Society Open Science* 6: 190368. 10.1098/rsos.190368. 31824687 10.1098/rsos.190368 PMC6837233

